# Fabrication of molecular tension probes

**DOI:** 10.1016/j.mex.2016.03.008

**Published:** 2016-03-18

**Authors:** Sung Bae Kim, Rika Fujii

**Affiliations:** Research Institute for Environmental Management Technology, National Institute of Advanced Industrial Science and Technology (AIST), 16-1 Onogawa, Tsukuba 305-8569, Japan

**Keywords:** Molecular tension probe, Luciferase, Bioluminescence, Molecular tension, Protein–protein interactions (PPI), *Renilla* luciferase, Bioluminescence imaging (BLI), Estrogen receptor

## Abstract

A unique bioluminescent imaging probe is introduced for illuminating molecular tension appended by protein–protein interactions (PPIs) of interest. A full-length luciferase is sandwiched between two proteins of interest via minimal flexible linkers. The ligand-activated PPIs append intramolecular tension to the sandwiched luciferase, boosting or dropping the enzymatic activity in a quantitative manner. This method guides construction of a new lineage of bioassays for determining molecular tension appended by ligand-activated PPIs.

The summary of the method is:

• Molecular tension appended by protein–protein interactions (PPI) is visualized with a luciferase.
• Estrogen activities are quantitatively illuminated with the molecular tension probes.
• Full-length *Renilla* luciferase enhances the optical intensities after bending by PPI.

## Method details

To date, several potential techniques have been established for determining protein–protein interactions (PPIs), including (i) Bioluminescence resonance energy transfer (BRET) based on energy transfer between bioluminescent donor and fluorescent acceptor proteins [1], [2], [3]; (ii) Mammalian/yeast two-hybrid assay reflecting interactions between “Prey” and “Bait” proteins [4]; (iii) Protein-fragment complementation assay (PCA) making use of split-reporter protein and its conditional reconstitution [5], [6].

We previously developed a unique bioluminescent probe called “strain probe” for illuminating PPIs [7]. We initially hypothesized that any luciferase has talent to change its enzymatic activity according to the molecular tension artificially appended by PPIs. This molecular tension may cause distortion of the active site, which modulates the enzymatic activity.

In this method, we introduce how to fabricate *molecular tension probe*s emitting bioluminescence in response to molecular tension appended by PPIs in detail.

### Materials

• pcDNA 3.1(+) (Invitrogen)
• a mammalian expression vector
• Restriction enzymes (*Hind*III, *BamH*I, *Kpn*I, *Xho*I)
• African green monkey kidney fibroblast-derived COS-7 cells
• A 96-well clear bottom microplate (Nunc)
• Dulbecco’s modified Eagle’s medium (DMEM)
• Fetal bovine serum (FBS; Gibco)
• Penicillin/streptomycin (P/S; Gibco)
• TransIT-LT1 (Mirus), a lipofection reagent
• 17β-Estradiol (E_2_; native estrogen)
• 4-Hydroxytamoxifen (OHT; synthetic antiestrogen)
• Phosphate-buffered saline (PBS)
• A lysis buffer (E291A, Promega)
• An assay buffer (E290B, Promega)
• Native coelenterazine (nCTZ)
• A Bradford reagent for determining total protein amounts
• Hank’s buffered salt solution (HBSS) buffer (Gibco)

### Basic concept for designing *molecular tension probes*

**Fig. 1:**
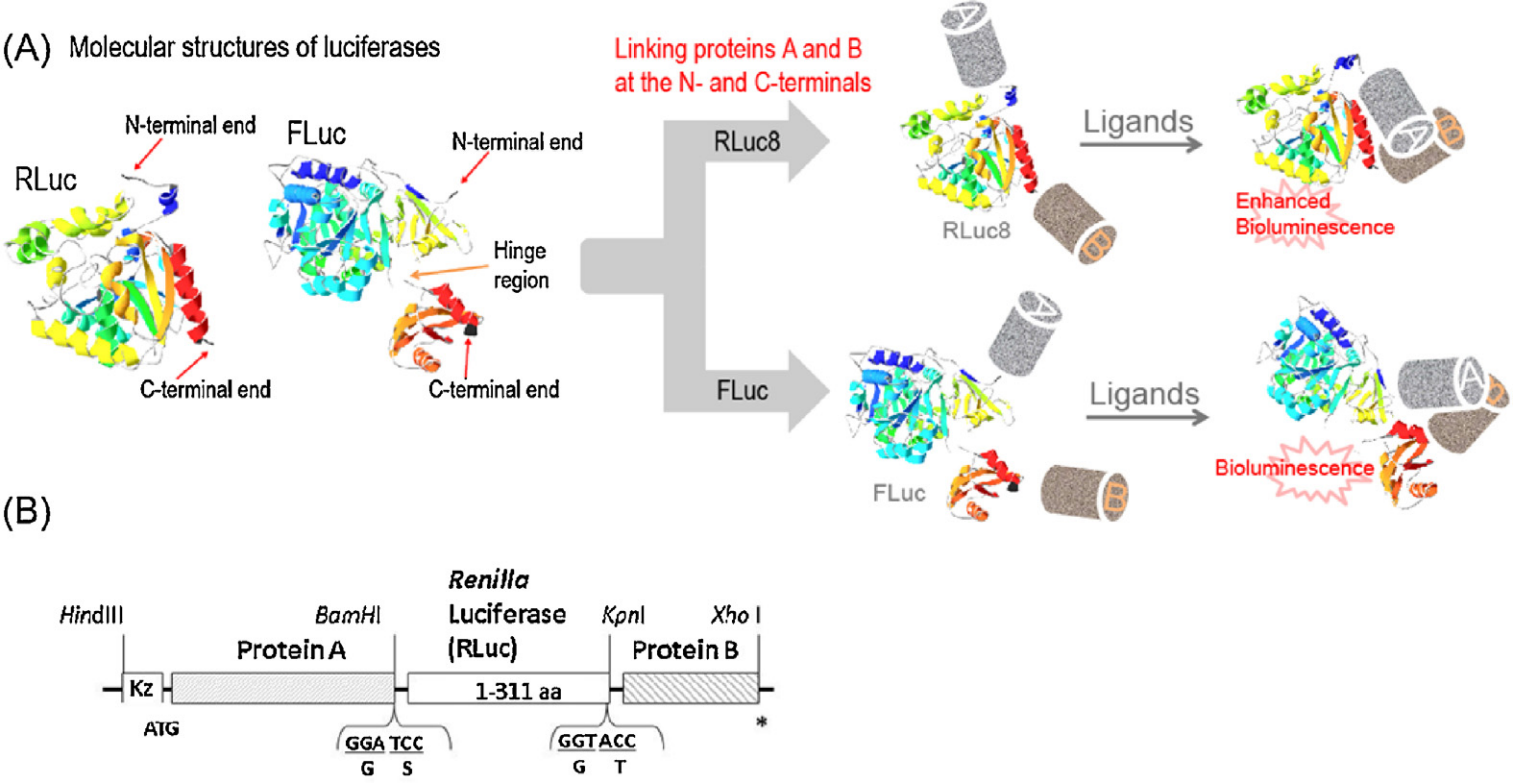
(A) The working mechanism of *molecular tension probes*. Any luciferase may be sandwiched between two proteins A and B of interest. A ligand triggers an interaction between proteins A and B, which appends tense to the sandwiched luciferase. (B) A schematic diagram of the cDNA construct showing the segments. The linkers between the segments were minimized. It was designed that the restriction sites are the only linkers to connect each segment in the construct. Abbreviations: RLuc8, A *Renilla* luciferase variant carrying 8 mutations; FLuc, a firefly luciferase; Kz, kozak sequence; ER LBD, the ligand binding domain of estrogen receptor; SH2, the Src homology domain 2 of *v*-Src.

The basic design of *molecular tension probes* consists of four different ingredients, i.e., a full-length luciferase, a pair of proteins of interest (called proteins “A” and “B”), and a flexible linker, where the luciferase is sandwiched between the two proteins of interest via a minimal length of flexible linkers (Fig. 1). The luciferase is tensed by the ligand-activated PPIs. The minimal flexible linkers as possible connecting the ingredients are advantageous to efficiently convert the molecular tension to practical distortion of the sandwiched luciferase.

Any luciferases basically have talent to vary their enzymatic activity more or less according to the molecular tension appended by an intra-molecular PPI. A globular marine luciferase may be advantageous over beetle luciferases, which consist of N- and C-terminal domains connected by a flexible hinge region [8] (Fig. 1A). A globular marine luciferase like *Renilla reniformis* luciferase (RLuc) easily receives tension from PPIs, whereas the flexible region in beetle luciferases relaxes the intra-molecular tension.^1^ The active site of RLuc8 is close from the C-terminal end [10], thus is prone to be influenced by protein-tagging and molecular tension appended by adjacent proteins.

In this protocol, we exemplify a *molecular tension probe* that is made of RLuc8 as a model luciferase sandwiched between the ligand-binding domain of the human estrogen receptor (ER LBD) as an intracellular receptor member of the nuclear receptor superfamily and Src homology domain 2 of *ν*-Src (SH2), based on our previous papers [7], [11]. Upon ligand activation, Tyr537 of ER LBD is phosphorylated and recognized by the counterpart SH2 domain. This molecular tension varies the bioluminescence intensity of the sandwiched RLuc8 (Fig. 1). The corresponding positive and negative control studies in a tension-free condition were well discussed in the original paper [7].

### Preparation of the cDNA constructs encoding a *molecular tension probe*

The cDNA constructs encoding *molecular tension probes* is fabricated by conventional genetic engineering techniques including polymerase chain reaction (PCR) with an adequate primer set and its subcloning into a mammalian expression vector as follow (Fig. 1).

#### Procedure

1. Generate the cDNA segments encoding full-length *Renilla* luciferase 8 (1–311 aa; RLuc8) by PCR using a corresponding primer set flanked with unique restriction sites, *BamH*I and *Kpn*I, for introducing unique restriction sites at the 5′- and 3′-terminals, respectively.
2. Fabricate the cDNA segments encoding the ER LBD and the SH2 domain of *v*-Src by PCR using corresponding primer sets flanked with the unique restriction sites, *Hind*III/*BamH*I, and *Kpn*I/*Xho*I, respectively.
3. Digest the above cDNA segments and the multiple cloning site (MCS) of a mammalian expression vector pcDNA 3.1(+) (Invitrogen) with the corresponding restriction enzymes, *Hind*III/*BamH*I; *BamH*I/*Kpn*I; *Kpn*I/*Xho*I; *Hind*III/*Kpn*I, respectively (Fig. 1B).^2^
4. Gel-purify the digested cDNA segments and the mammalian expression vector.
5. Ligate the cDNA segments into the expression vector *p*cDNA 3.1(+) (Invitrogen) to fabricate a cDNA construct encoding the *molecular tension probe* (single coding sequence) (Fig. 1B).
6. Confirm the sequences of the cDNA constructs in pcDNA3.1(+) vector with a genetic sequencer (GenomeLab GeXP, Beckman Coulter) (named *p*Ers).^3^

### Bioluminescence spectra of COS-7 cells carrying pErs

**Fig. 2:**
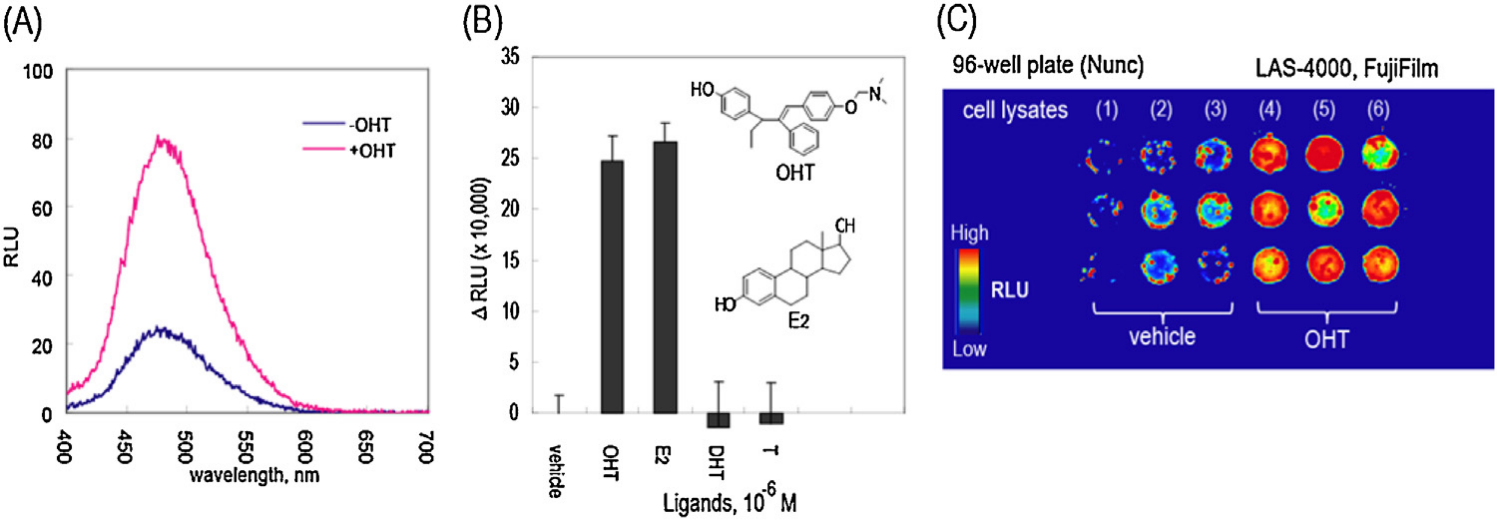
(A) Variance in the bioluminescence emission spectra before and after addition of 1 μM 4-hydroxytamoxifen (OHT). The figure was modified from our previous study [7]. (B) Ligand selectivity of ERS. The luminescence intensities were compared after activation of ERS with various ligands. The figure was modified from our previous study [7]. (C) An optical image of the lysates of COS-7 cells after stimulation with vehicle or OHT (n = 9).

The ligand-driven variance of optical spectra can be determined with a mammalian culture cell line, COS-7 cells, expressing *the molecular tension probe* as follows (Fig. 2A).

#### Procedure

1. Grow COS-7 cells derived from African green monkey kidney fibroblast^4^ in a Dulbecco’s modified Eagle’s medium (DMEM) supplemented with 10% fetal bovine serum (FBS; Gibco), and 1% penicillin/streptomycin (P/S; Gibco) at 37 °C in a 5% CO_2_ incubator.
2. Seed the cells in 12-well culture plates, transiently transfected with an aliquot (1 μg) of *p*Ers per well using a transfection reagent, TransIT-LT1 (Mirus).
3. Further, incubate the cells for 16 h in the cell incubator (5% CO_2_, 37 °C) before bioluminescence measurement.^5^
4. Stimulate the cells on the 12-well plates for 20 min with the vehicle (0.1% DMSO), 1 μM of 17β-estradiol (E2; agonist) or 1 μM of 4-hydroxytamoxifen (OHT; antagonist). Wash the cells once with phosphate-buffered saline (PBS; pH 7.4, 0.02 M).
5. Lyse the cells with a lysis buffer (E291A, Promega) for 15 min in room temperature.
6. Transfer an aliquot (10 μL) of the lysates into a 200 μL microtube and determine the bioluminescence spectra with a spectrophotometer^6^ immediately after addition of an aliquot (50 μL) of an assay buffer (E290B, Promega) dissolving native coelenterazine (nCTZ) into the microtube.

### Determination of estrogenic activity of chemicals with a *molecular tension probe*

Estrogenicity of steroidal hormones and synthetic chemicals is visualized with a *molecular tension probe*, ERS, as follows (Fig. 2B).

#### Procedure

1. Grow COS-7 cells in a 96-well clear-bottom microplate until a 70% fluency in the cell incubator (5% CO_2_, 37 °C).
2. Transiently transfect the COS-7 cells in the wells of the microplate with an aliquot of *p*Ers (0.2 μg per well) using the lipofection reagent (TransIT-LT1, Mirus) and incubate them in the cell incubator for 16 h.
3. Stimulate the cells with the vehicle, 10^−6^ M of E_2_, or 10^−6^ M of OHT for 20 min.^7^
4. Wash the cells once in the plate with PBS, and lyse them with a lysis buffer (E291A, Promega) for 15 min.
5. Transfer the cell lysates to a 1.6 mL microtube and determine the subsequent bioluminescence intensities with a luminometer (GloMax 20/20, Promega) immediately after adding an assay solution (E290B, Promega) dissolving nCTZ.
6. Parallelly, measure the total protein amounts in the aliquot of the cell lysates with a Bradford reagent for the following normalization procedure.
7. Normalize the RLuc luminescence intensities from Step 5 in a ratio of relative luminescence unit (RLU), that is, RLU (+)/RLU (−), where RLU (+) and RLU (−) are the luminescence intensities with 1 μg of cell lysate (Step 6) after the cells were incubated with and without a ligand, respectively, where the RLU is an amplified value of photon counts generated from the luminometer (arbitrary unit) (Fig. 2B).

The procedure from Steps 5 to 7 may be substituted by the following alternative (Fig. 2C).

5a. Transfer an aliquot of the cell lysates (10 μL) to each well of a fresh 96-well optical bottom plate.^8^

6a. Simultaneously inject an aliquot (50 μL) of the assay buffer dissolving nCTZ into the cell lysates on the plate using a multichannel pipette^9^ and immediately transfer the plate in an image analyzer (LAS-4000, FujiFilm).

7a. Determine the optical intensities in the plate with the equipped controlling software (Image reader ver2.0) and analyze the optical images with the analysis software (Multi Gauge ver3.1).

8a. Normalize the optical intensities (originally expressed in RLU/mm^2^) to the integrated time (second) and applied protein amounts (μg): i.e., RLU/μg/sec/mm^2^.

### Bioluminescence imaging (BLI) of living mammalian cells with a *molecular tension probe*

**Fig. 3:**
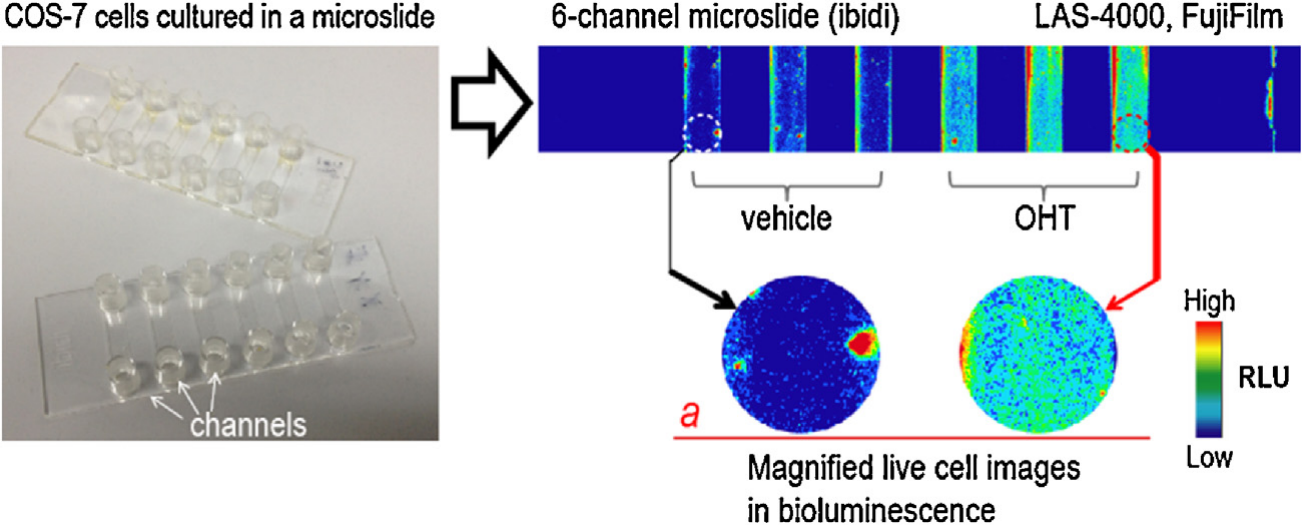
A bioluminescence image (BLI) of COS-7 cells carrying the tension probe on a microslide (μ-Slide IV^0.4^, ibidi). The 3 channels on the left and right are stimulated with vehicle and ligand, respectively. The experiment was conducted in triplicate (n = 3). The inset *a* shows a magnified optical image of the microslide.

Live-cell images by the tension probe are conducted with the following procedure (Fig. 3).

#### Procedure

1. Grow COS-7 cells in a 6-channel microslide (μ-slide VI^0.4^, ibidi) until a 70% fluency.^10^
2. Transiently transfect the cells in the channels of the microslide with *p*Ers using a lipofection reagent (TransIT-LT1, Mirus) and keep them in a cell incubator (5% CO_2_, 37 °C) for 16 h.
3. Stimulate the cells in the channels with vehicle (0.1% DMSO) or 10^−6^ M OHT for 20 min before image acquisition.
4. Wash the cells in the channels once with a Hank’s buffered salt solution (HBSS) buffer (Gibco) and then fill the channels simultaneously with 80 μL of an HBSS buffer dissolving nCTZ using a multichannel pipet.
5. Immediately transfer the microslide into an image analyzer (LAS-4000mini, FujiFilm) and determine the optical images with the equipped software (Image reader ver2.0 and Multi Gauge ver3.1).

## Footnotes

1 The C-terminal domain of firefly luciferase (FLuc) is rotated to release luciferin in the light-emitting process [9], which is hampered by the molecular tension in the probe.

2 A double digestion is convenient with the Cutsmart buffer of New England Biolab.

3 In this protocol, the made probes are named “ERS” from the consecutive initial letters of the components, i.e., ER LBD-RLuc8-SH2. The corresponding plasmid was named *p*Ers.

4 Any mammalian cell lines are basically applicable for this protocol.

5 The incubation time influences on the signal-to-background (S/B) ratios. A better S/B ratio is achieved by avoiding the overexpression.

6 Recommended is a spectra photometer equipped with a cooled CCD camera which simultaneously acquires the whole range of wavelength, instead of scanning the wavelength range.

7 A concentration ranging from10^−5^ to 10^−7^ M of steroid is appropriate for minimizing the background intensities.

8 A microplate manufactured for bioluminescence is adequate for this protocol.

9 8- or 12-channel pipettes are useful for the simultaneous injection.

10 Because of the small channel volume of the slide, the culture medium is prone to be condensed by evaporation. The culture medium needs to be refreshed every 12 h for minimizing the evaporation influence to the cells.
